# Infrared Brazing of CoCrFeMnNi Equiatomic High Entropy Alloy Using Nickel-Based Braze Alloys

**DOI:** 10.3390/e21030283

**Published:** 2019-03-15

**Authors:** Chieh Lin, Ren-Kae Shiue, Shyi-Kaan Wu, Huai-Li Huang

**Affiliations:** 1 Department of Materials Science and Engineering, National Taiwan University, Taipei 106, Taiwan; 2 Department of Mechanical Engineering, National Taiwan University, Taipei 106, Taiwan

**Keywords:** high entropy materials, infrared brazing, nickel-based filler metal, microstructure

## Abstract

Infrared vacuum brazing of CoCrFeMnNi high entropy alloy (HEA) using BNi-2 and MBF601 fillers has been investigated. Both brazes show poor wettability at temperatures only 20 °C above their liquidus temperatures. However, the wettability of BNi-2 and MBF601 fillers on CoCrFeMnNi HEA is greatly improved with increasing the test temperatures, 50 °C above their liquidus temperatures. The BNi-2 brazed joints are dominated by Ni-rich matrix with huge CrB and a few tiny boride precipitates. Average shear strengths of joints increase with increasing brazing temperature and/or time, and fracture location changes from blocky CrB in the brazed zone to grain boundary boride in the substrate. The MBF601 brazed joints are composed of CoCrFeMnNi-based matrix, particles of B/Co/Cr/Fe/Mn/Ni/P compounds, and some phosphides form along the grain boundaries of the substrate. The specimen brazed with MBF601 filler foil at 1050 °C for 600 s has the highest average shear strength of 321 MPa, while that brazed at 1080 °C for 600 s has a lower average shear strength of 271 MPa due to the presence of solidification shrinkage voids.

## 1. Introduction

High entropy alloys (HEAs) are defined as alloys containing at least five elements as the principal alloying ingredients, with each element in amounts of 5 to 35 at.% [1,2]. Numerous studies have shown that HEAs have many outstanding and attractive properties because they have the following four core effects: the high entropy effect, the sluggish diffusion effect, the severe lattice distortion effect, and the cocktail effect [2,3]. The equiatomic CoCrFeMnNi HEA was first reported by Cantor et al. [4] and was one of the most popular HEAs because it was found to be a face-centered cubic (FCC) single phase below melting point [5,6]. The CoCrFeMnNi HEA has good strength, ductility, formability, toughness at low temperature [7,8,9]. It is a potential candidate applied to heat exchanger for cryogenic application.

Joining of HEAs is important in application of such new alloys. For example, advanced brazing technology applied in joining the HEA could be crucial in developing novel corrosion-resistant heat exchanger made by HEA. Infrared heating is characterized with high heating rate up to 50 °C/s, which is much faster than that of traditional furnace brazing [10,11]. With the use of infrared brazing, the very early stage reaction at the interface of the substrate and the filler can be examined, and the mechanism of the early stage kinetics in brazing can thereby be revealed. In previous studies, infrared vacuum brazing has been used to examine the similar/dissimilar joining [12,13]. Experimental results indicate that infrared brazing can be used to accurately reveal the early stage evolution and kinetics of the brazed joints. 

In a previous study [14], NiMnFeCoCu HEA was used as a filler metal to braze Inconel 718 superalloy and showed good mechanical performance. However, no studies have reported the use of a filler metal to braze HEA. In this study, infrared brazing was used to braze CoCrFeMnNi HEA with two nickel-based braze alloys, BNi-2 and MBF601 filler metals, to unveil the early stage kinetics in brazing HEA. The wettability, microstructural evolution and shear strength of the brazed joints under different brazing conditions are investigated.

## 2. Materials and Methods

A CoCrFeMnNi ingot of approximately 100 g was prepared by vacuum arc remelter (VAR) with high purity raw materials of cobalt, chromium, iron, nickel and the master alloy of nickel-manganese (50-50 in wt%). Nickel-manganese master alloy of 99.9 wt% purity was used to reduce the evaporation of manganese in arc remelting. The purity of the rest raw materials was above 99.9 wt%. The ingot was remelted six times in high purity argon atmosphere and subsequently homogenized at 1200 °C for 24 h. The ingot was cold-rolled into a plate of 3.5 mm thickness with 50% reduction in thickness at room temperature. Specimens of 15 mm × 7 mm × 3.5 mm were cut from the cold-rolled plate by wire electrical discharge machining for microstructural observations, wetting angle measurements and shear tests. The brazing surfaces of the specimens were ground with SiC papers up to 1200 grit. BNi-2 and MBF601 filler foils of 50 and 38 μm thickness, respectively, were purchased from Prince & Izant Company (Cleveland, OH, USA) and Metglas Inc. (Conway, SC, USA), respectively. The compositions and melting ranges of these filler foils are listed in Table 1 [15].

The dynamic wetting angles of the filler metals on the CoCrFeMnNi substrate were measured by a sessile drop test. BNi-2 and MBF601 filler balls weighing approximately 0.15 g were prepared by VAR. Schematic diagrams of the wetting angle measurement equipment can be found in previous studies [16]. An infrared furnace (ULVAC SINKU-RIKO RHL-P816C, Tokyo, Japan) with vacuum of 5 × 10^−5^ mbar was applied for dynamic wetting angle measurement. The filler ball was placed on the CoCrFeMnNi substrate with a thermocouple in contact with the substrate during infrared brazing. The test conditions used in the dynamic wetting angle measurements were 1020 °C and 1050 °C for the BNi-2 filler and 1050 °C and 1080 °C for the MBF601 filler, and all brazing times were 300 s. The temperatures used in this study were the temperatures 20 °C and 50 °C above their liquidus temperatures.

**Figure 1:**
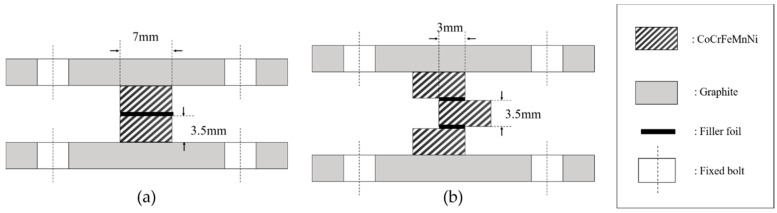
Schematic diagrams of brazed specimens used for (**a**) microstructural observation and (**b**) shear test.

An infrared furnace with a heating rate of 15 °C/s was used to braze all specimens for microstructural observations and shear tests. During the infrared brazing, a vacuum of 5 × 10^−5^ mbar was maintained in the furnace. The brazing conditions for the shear tests, microstructural observations and dynamic wetting angle measurements are shown in Table 2. All specimens were preheated at 900 °C for 300 s to equilibrate the specimens’ temperatures. Figure 1a,b illustrate the schematic diagrams of the specimens enclosed in graphite fixtures for microstructural observations and shear tests, respectively.

The quantitative chemical compositions of brazed specimens were examined with an electron probe microanalyzer (EPMA) (JXA-8200, JEOL, Tokyo, Japan) equipped with a wavelength dispersive spectroscope (WDS). Shear tests were performed with a universal tensile test machine (AG-IS, Shimadzu Crop., Kyoto, Japan) with a constant compressive strain rate of 0.0167 mm/s. For each brazing condition, three brazed specimens were shear-tested to obtain the average shear strength. The fractured surfaces and cross-sections of shear test specimens were cut by a low-speed diamond saw and observed with a field emission scanning electron microscope (FESEM, NOVA NANO 450, FEI Crop., Hillsboro, OR, USA).

## 3. Results and Discussion

### 3.1. Dynamic Wetting Angle Measurement

**Figure 2:**
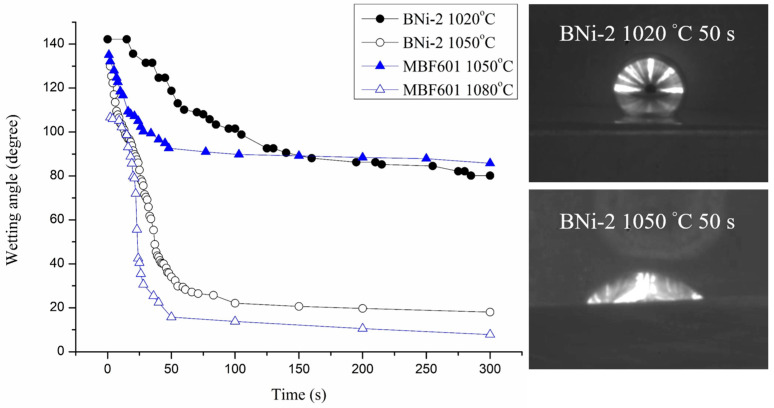
Dynamic wetting angle measurements and selected images of molten BNi-2 braze on CoCrFeMnNi substrate.

A dynamic wetting angle measurement experiment was carried out in 0–300 seconds at different temperatures. Figure 2 shows the results of dynamic wetting angle measurements and selected images of the BNi-2 filler heated at 1020 °C and 1050 °C for 50 s on the CoCrFeMnNi substrate. The wetting angle was obtained from the automatic image analysis software. For example, the wetting angle is averaged from left and right angles obtained from the image shown in Figure 2. The accuracy of the dynamic wetting angle measurements primarily depends on the quality of captured image. If the captured image is as clear as displayed in Figure 2, accuracy of the wetting angle will be below 1°. For the BNi-2 filler metal heated at 1020 °C, the wetting angle is 142 degrees at first and falls slowly to 80 degrees at 300 s. For the BNi-2 filler metal heated at 1050 °C, the wetting angle drops quickly from 132 degrees to 43 degrees in the first 40 s and then falls slowly to 18 degrees at 300 s. For the MBF601 filler metal heated at 1050 °C, the wetting angle is 137 degrees at first and falls slowly to 86 degrees at 300 s. For the MBF601 filler metal heated at 1080 °C, the wetting angle drops quickly from 112 degrees to 40 degrees after 25 s and then falls slowly to 8 degrees at 300 s. The dynamic wetting angle is related to the surface tension of molten metal on a substrate. As shown in Figure 2, the BNi-2 and MBF601 filler metals both exhibit excellent wettability on the CoCrFeMnNi substrate at temperatures 50 °C above their liquidus temperatures, but they show poor wettability at temperatures only 20 °C above their liquidus temperatures. 

The wettability study of BNi-2 braze on 422 stainless steel using the traditional furnace heating has been reported [17]. Similar wetting behavior was observed. The BNi-2 shows poor wetting at 1025 °C. However, the wettability is greatly improved at 1050 °C for 600 s. The dynamic wetting angle is related to the surface tension of brazed melt and/or interfacial reaction(s) between the braze melt and substrate. Increasing the test temperature can decrease the surface tension between the molten braze and substrate [18]. Meanwhile, increasing the test temperature can also enhance interfacial reaction(s). Reactive wetting of CoCrFeMnNi HEA substrate by BNi-2 and MBF601 brazes could be attributed to the formation of interfacial/intergranular boride and/or phosphide. They all improve wetting angles of the braze melt on CoCrFeMnNi substrate. BNi-2 and MBF601 fillers are categorized as Ni-based braze alloys. It is suitable for brazing most Fe/Ni/Co-based alloys [18,19]. Because Fe, Ni and Co are the major ingredients in the Cantor alloy, it is reasonable that excellent wettability on the Cantor alloy is obtained using BNi-2 and MBF601 brazes.

### 3.2. Microstructural Observations of Infrared Brazed CoCrFeMnNi/BNi-2/CoCrFeMnNi Joints

**Figure 3:**
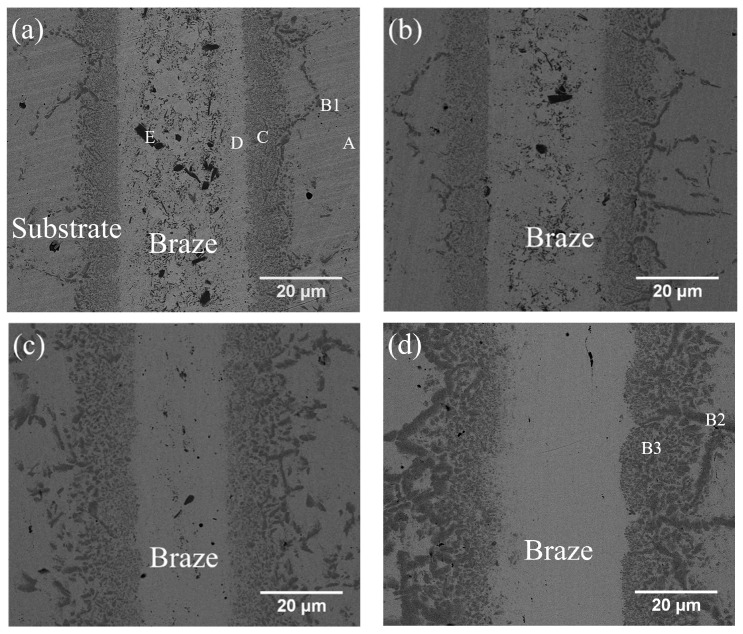
SEM BEI cross-sections of CoCrFeMnNi/BNi-2/CoCrFeMnNi joints infrared brazed at (**a**) 1020 °C for 180 s, (**b**) 1020 °C for 300 s, (**c**) 1020 °C for 600 s and (**d**) 1050 °C for 600 s.

**Figure 4:**
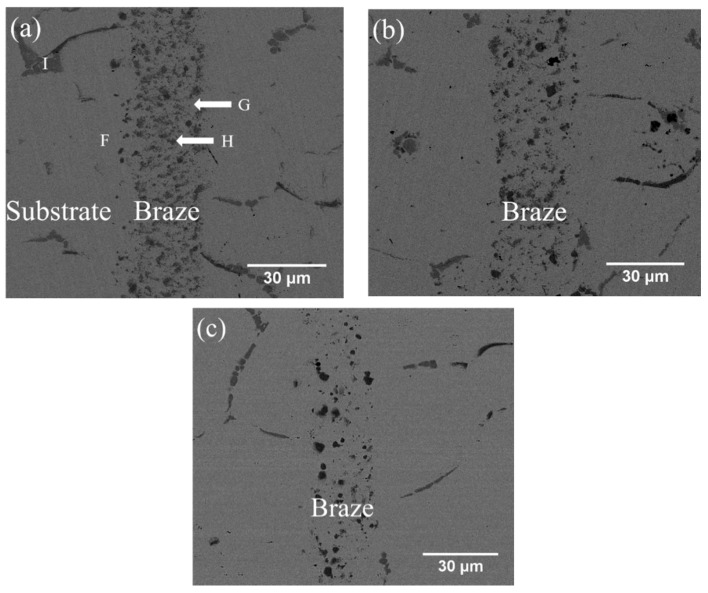
SEM BEI cross-sections of CoCrFeMnNi/MBF601/CoCrFeMnNi joints infrared brazed at (**a**) 1050 °C for 180 s, (**b**) 1050 °C for 600 s, and (**c**) 1080 °C for 600 s.

Figure 3a–d and Table 3 show the cross-sections of SEM backscattered electron images (BEIs) and the EPMA quantitative chemical analysis results of CoCrFeMnNi/BNi-2/CoCrFeMnNi joints brazed at 1020 °C for 180 s, 300 s, and 600 s and at 1050 °C for 600 s, respectively. In Figure 3a, the region marked A is the CoCrFeMnNi HEA substrate. The brazed joint consists of many large, irregularly-shaped CrB borides (marked E) in the Ni-rich matrix (marked D). Figure 3a also shows many tiny particles in the Ni-rich matrix, which are too small to identify with EPMA analysis (Table 3). They may have been silicide/boride precipitates, for the EPMA result indicates that the Ni-rich matrix (marked D) is alloyed with 5.4 at.% B, 7.6 at.% Si, 4.6 Fe, 6.4 Cr, 0.5 Mn and 1.3 Co. Figure 3a–d also show a layer between the substrate and Ni-rich matrix. This layer, marked C in Figure 4a, has many fine precipitates. It is mainly composed of B, Co, Cr, Fe, Ni and Mn, so these fine precipitates in layer C could be borides. This layer of mixed tiny borides and Ni-rich phase is referred to as the boride layer in the following text. It is noted that the stoichiometric ratio of the borides in this layer could not be accurately determined due to the lack of the related phase diagram.

Increasing the brazing time from 180 s to 300 s and then to 600 s decreases the number of borides in the central region of Ni-rich matrix. The thickness of the central Ni-rich matrix simultaneously decreases, as illustrated in Figure 3a–c. The disappearance of the central region borides in the Ni-rich matrix results from the depletion of boron atoms from the braze melt into the substrate. The boron atoms diffuse into the boride layer, increasing the layer thickness and the size of the boride precipitates. In the joint brazed at 1050 °C for 600 s (Figure 3d), CrB precipitates disappear entirely from the central region of the Ni-rich matrix, and the boride layer thickens. Both increasing the brazing time and increasing the temperature decrease the amount of CrB precipitates in the central brazed zone. At the same time, the boride layer between the brazed zone and the substrate coarsens due to the depletion of boron atoms from the braze melt into the substrate during brazing. 

It is worth mentioned that the depletion of B atoms from the brazed zone into the CoCrFeMnNi HEA substrate primarily relies on grain boundary diffusion. Grain boundary B reacts simultaneously with Co, Cr, Fe Mn and Ni, and forms grain boundary boride in the substrate next to the brazed zone. In Figure 3a,d and Table 3, B1, B2 and B3 have similar chemical composition, so they are classified as the boride phase. The boride phase is coarsened with increasing the brazing temperature and/or time due to enhanced grain boundary diffusion of B.

### 3.3. Microstructural Observations of Infrared Brazed CoCrFeMnNi/MBF601/CoCrFeMnNi Joints

Figure 4a–c and Table 4 show the cross-sections of SEM BEIs and the EPMA quantitative chemical analysis results of CoCrFeMnNi/MBF601/CoCrFeMnNi joints brazed at 1050 °C for 180 s and 600 s and at 1080 °C for 600 s, respectively. In Figure 4a, the region marked F is the substrate of CoCrFeMnNi HEA close to the joint. The brazed joint is composed of CoCrFeMnNi-based matrix, marked G, and particles of B/Co/Cr/Fe/Mn/Ni/P compounds, marked H. High B and P concentrations at the H point in Figure 4a and Table 4, so it is a compound, not a solid solution. Unfortunately, there is little information relating to such a compound with more than three elements. Figure 4a also presents phosphides along the grain boundaries of the substrate, marked I. According to the EPMA chemical analysis results in Table 4, the HEA substrate (marked F) has Fe and Ni contents higher than those of the equiatomic HEA due to the high Fe and Ni contents of the MBF601 filler foil. As shown in Figure 4a–c, the microstructures are relatively similar in samples brazed for 180 s or 600 s at 1050 °C, and the number of particles of B/Co/Cr/Fe/Mn/Ni/P compounds in the joint is higher after brazing at 1050 °C than after brazing at 1080 °C.

Mass transport during brazing involves two important mechanisms, dissolution of HEA substrate into the molten braze and diffusion of the braze alloy into substrate. Additionally, potential chemical reaction(s) among selected elements must also be considered. For BNi-2 and MBF601 fillers, transports of B or P play important roles in brazing, since huge amounts of borides and phosphides are observed in the brazed joint (Figure 3 and Figure 4). The formation of these compounds not only strongly affects distribution of various elements across the joint but also bonding strength of the joint.

### 3.4. Shear Strength and Failure Analyses of Fractured Surfaces after Shear Tests

Table 5 shows the average shear strengths of specimens infrared brazed under different conditions. The average shear strengths of the joints brazed with BNi-2 filler metal at 1020 °C for 180 s, 300 s, and 600 s and at 1050 °C for 600 s are 193, 305, 310 and 319 MPa, respectively. Yield strength of annealed CoCrFeMnNi HEA at room temperature is strongly related to its grain size, and the Hall-Petch relationship is fitted well from the ultrafine-grained regime to the coarse-grained regime [20,21]. The grain size of CoCrFeMnNi HEA is approximately 30~100 μm, which belongs to the category of coarse grained region. The yield strength of CoCrFeMnNi HEA at room temperature with the grain size of 30–100 μm is between 200–250 MPa depending on the thermal history of the alloy [20,21,22,23]. Based on the von Mises criterion, the ratio between shear strength and tensile yield strength is 0.577, (1/$\sqrt{3}$) [24]. The estimated equivalent shear strength is 115–144 MPa if the tensile yield strength is between 200 and 250 MPa. Average shear strength of the brazed joint exceeding 193 MPa is higher than the estimated equivalent shear strength of CoCrFeMnNi HEA substrate. It is demonstrated by the presence of severe deformed CoCrFeMnNi HEA substrate after shear test. Therefore, BNi-2 and MBF601 show potential in brazing CoCrFeMnNi HEA.

**Figure 5:**
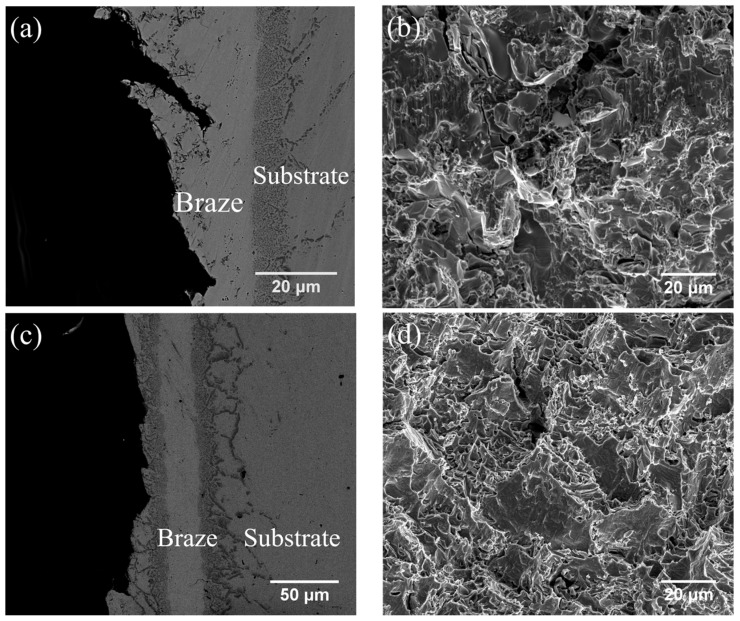
SEM (**a**) BEI cross-section, (**b**) SEI fractograph of CoCrFeMnNi/BNi-2/CoCrFeMnNi joint infrared brazed at 1020 °C for 180 s, (**c**) BEI cross-section and (**d**) SEI fractograph of CoCrFeMnNi/BNi-2/CoCrFeMnNi joint infrared brazed at 1050 °C for 600 s.

Figure 5a,b and Figure 5c,d show the cross-sections and fractured surfaces of the specimens infrared brazed at 1020 °C for 180 s and at 1050 °C for 600 s, respectively. The specimen infrared brazed at 1020 °C for 180 s shows the lowest average shear strength of 193 MPa. From Figure 5a, one can find that cracks initiated and propagated in the central brazed zone, and cleavage dominated fractograph as illustrated in Figure 5b is observed. It is deduced that the existence of black blocky CrB, marked by E in Figure 3a, deteriorates shear strength of the infrared brazed joint. According to Figure 3, the amount of brittle blocky CrB compound decreases with increasing the brazing time and/or temperature due to depletion of the B content from the brazed zone into CoCrFeMnNi HEA substrate. The joint infrared brazed at 1050 °C for 600 s is free of brittle CrB compound as displayed in Figure 3d, and it demonstrates the highest average shear strength of 319 MPa. In Figure 5c, the crack initiates from a boride layer and grain boundary boride as made by B3 and B2 in Figure 3d, respectively. Figure 5d shows the secondary electron image (SEI) fractograph of the joint infrared brazed at 1050 °C for 600 s after shear test. It is characterized with quasicleavage fracture of large grain boundary boride and fine boride in the layer.

**Figure 6:**
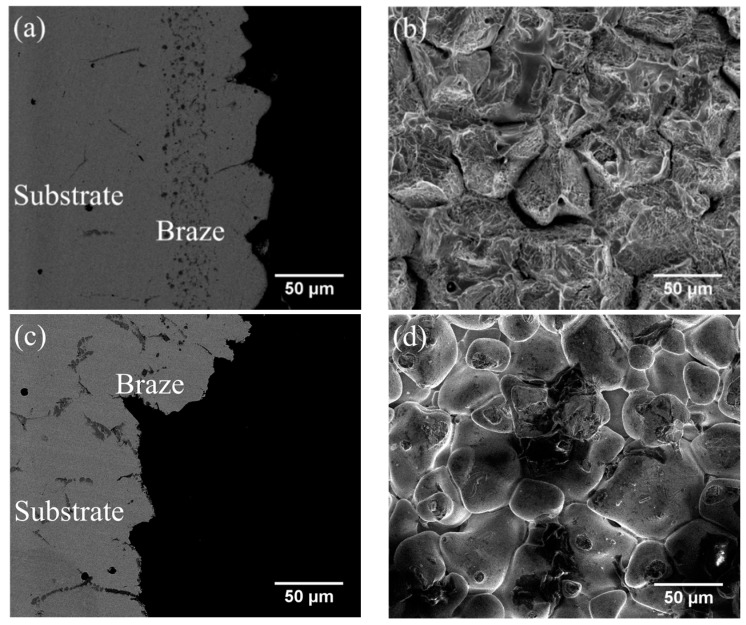
SEM (**a**) BEI cross-section, (**b**) SEI fractograph of CoCrFeMnNi/MBF601/CoCrFeMnNi joint infrared brazed at 1050 °C for 600 s, (**c**) BEI cross-section and (**d**) SEI fractograph of CoCrFeMnNi/MBF601/CoCrFeMnNi joint infrared brazed at 1080 °C for 600 s.

Table 5 also shows that the average shear strengths of the joints infrared brazed with MBF601 filler metal at 1050 °C for 180 s and 600 s and at 1080 °C for 600 s are 291, 321 and 271 MPa, respectively. Figure 6a,b show the cross-section and fractured surface of the joint infrared brazed at 1050 °C for 600 s, which has the highest average shear strength of 321 MPa. From Figure 6a, one can find that the fracture is located on the substrate side. In Figure 6b, brittle intergranular fracture is visible, as is the ductile dimple fracture in the grain. According to the EPMA chemical analysis results in Table 4, the intergranular fracture is caused by the brittle phosphides along the grain boundaries of the CoCrFeMnNi substrate. CoCrFeMnNi-based HEA is well known for its great ductility [7,8,9]; however, the presence of the phosphides in the substrate’s grain boundaries deteriorates the ductility of the joint. This deterioration is the main cause of joint failure in the shear test. 

**Figure 7:**
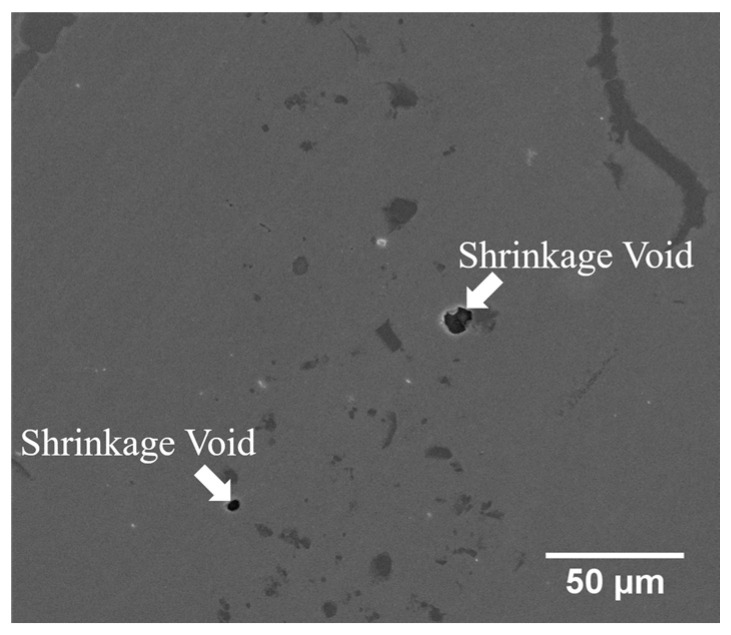
SEM SEI cross-section of CoCrFeMnNi/MBF601/CoCrFeMnNi joint infrared brazed at 1080 °C for 600 s.

Figure 6c,d display the SEM BEI cross-section and SEI fractograph of the joint brazed at 1080 °C for 600 s with the lowest average shear strength of 271 MPa. Different from Figure 6a, the fracture is located in the central brazed zone. According to Figure 4c, solidification shrinkage voids cannot be well observed in backscattered electron image (BEI) due to its insufficient topographic contrast. A secondary electron image (SEI) is included in Figure 7 in order to reveal solidification shrinkage voids in the cross section of the brazed joint. Figure 6d shows an SEI fractograph of the solidification shrinkage voids after shear test. Higher brazing temperature and time result in rapid depletion of melting point depressants, P and B, in the braze alloy [25]. The molten braze is prone to proceed isothermal solidification during brazing. Consequently, shrinkage voids are formed due to insufficient liquid in the brazed zone at the final stage of solidification.

According to Table 1, the difference between solidus and liquidus temperatures of BNi-2 is much less than that of MBF601. It indicates that the chemical composition of BNi-2 is much closer to eutectic than that of MBF601. The BNi-2 braze melt is characterized with higher fluidity, and shows better solidification behavior as compared with MBF601. Therefore, the presence of solidification shrinkage void is significantly decreased in the BNi-2 brazed joint.

## 4. Conclusions

The wettability, microstructural evolution, and shear tests of CoCrFeMnNi equiatomic HEA infrared brazed with BNi-2 and MBF601 filler metals are investigated. Important findings are summarized as follows:

1. The dynamic wetting angle measurement indicates that the wettability of BNi-2 and MBF601 filler metals is great at the temperatures 50 °C above their liquidus temperatures, but poor at the temperatures 20 °C above their liquidus temperatures.
2. CoCrFeMnNi/BNi-2/CoCrFeMnNi brazed joints are dominated by Ni-rich matrix with huge CrB and a few tiny boride precipitates. Increasing the brazing time and/or temperature decrease the number of borides in the joint, and the grain boundary boride in the substrate becomes thicker and coarser. Average shear strengths of joints increase with increasing brazing temperature and/or time, and fracture location is changed from blocky CrB in the brazed zone into grain boundary boride in the substrate.
3. CoCrFeMnNi/MBF601/CoCrFeMnNi brazed joints are composed of CoCrFeMnNi-based matrix, particles of B/Co/Cr/Fe/Mn/Ni/P compounds, and many phosphides form along the grain boundaries of the substrate. The specimen brazed with MBF601 filler foil at 1050 °C for 600 s has the highest average shear strength of 321 MPa, while that brazed at 1080 °C has a lower average shear strength due to the presence of solidification shrinkage voids.

## Figures and Tables

**Table 1:** Compositions and melting temperatures of two brazing filler foils [15].

Filler Foil	BNi-2	MBF601
Composition (wt%)	Ni-7Cr-3.125B-4.5Si	Ni-16Cr-32Fe-1.5Si-0.5B-6P-1.5Mo
Solidus temperature	971 °C	960 °C
Liquidus temperature	998 °C	1030 °C

**Table 2:** Summary of brazing conditions used in the experiment.

Filler Foil	Brazing Time (s)	Brazing Temperature (°C)		
		1020	1050	1080
BNi-2	180	S/M		
	300	S/M/W	W	
	600	S/M	S/M	
MBF601	180		S/M	
	300		W	W
	600		S/M	S/M

S: Shear test, M: Metallographic observation, W: Dynamic wetting angle measurement.

**Table 3:** Electron probe microanalyzer (EPMA) chemical analysis results in Figure 3.

at.%	A	B1	B2	B3	C	D	E
Co	20.2	7.8	6.6	6.0	19.0	1.3	0.1
Cr	20.5	37.0	42.0	46.2	19.3	6.4	46.5
Fe	20.0	10.3	9.7	9.3	17.9	4.6	0.4
Mn	19.8	11.6	10.8	10.5	14.4	0.5	0.3
Ni	19.5	5.9	4.3	3.9	21.4	74.2	7.6
B	-	27.4	26.6	24.1	7.6	5.4	44.5
Si	-	-	-	0.1	0.4	7.6	0.6
Phase	CoCrFeMnNi	boride	boride	boride	-	Ni-rich	CrB

**Table 4:** EPMA chemical analysis results in Figure 4a.

at.%	F	G	H	I
Co	17.7	11.8	7.6	12.6
Cr	19.2	19.5	34.5	25.8
Fe	22.9	24.5	10.1	7.2
Mn	15.1	12.8	8.3	11.5
Ni	23.7	26.3	9.4	10.1
B	-	2.4	13.1	1.5
Si	0.5	1.0	0.2	0.1
P	0.8	1.4	15.9	30.9
Mo	0.1	0.3	0.9	0.3
Phase	CoCrFeMnNi-based	CoCrFeMnNi-based	B/Co/Cr/Fe/Mn/Ni/P compound	Phosphide

**Table 5:** Average shear strength of infrared brazed specimens with different conditions.

Filler Foil	Brazing Temperature (°C)	Brazing Time (s)	Average Shear Strength (MPa)
BNi-2	1020	180	193 ± 21
		300	305 ± 27
		600	310 ± 47
	1050	600	319 ± 21
MBF601	1050	180	291 ± 28
		600	321 ± 28
	1080	600	271 ± 6
